# Oncostatin M silence and neopeptide: the value of exploring patients with rare inherited bone marrow failure

**DOI:** 10.1172/JCI190955

**Published:** 2025-03-17

**Authors:** Selket Delafontaine, Isabelle Meyts

**Affiliations:** 1 Laboratory for Inborn Errors of Immunity, Department of Microbiology, Immunology and Transplantation, KU Leuven, Leuven, Belgium.; 2 Department of Pediatrics, University Hospitals Leuven, Leuven, Belgium.

## Abstract

Inherited bone marrow failure syndromes (IBMFSs) encompass a diverse group of hematological disorders characterized by a progressive single-lineage cytopenia or pancytopenia. Despite their heterogeneity, these syndromes often result from genetic errors affecting key biological mechanisms, including telomere maintenance, DNA repair and chromosomal stability, and ribosome assembly, generally leading to accelerated apoptosis of hematopoietic cells. Nevertheless, a genetic diagnosis remains elusive in more than half of the cases. The increased risk of myelodysplastic syndrome (MDS), acute leukemia, and solid tumors associated with IBMFS frequently prompts early hematopoietic stem cell transplantation (HSCT). In this issue of the *JCI*, Garrigue, Kermasson, and colleagues identified a homozygous variant in Oncostatin M (*OSM*) in 3 children from a consanguineous family presenting with IBMFS characterized by profound anemia, thrombocytopenia, and neutropenia. The findings suggest that the loss-of-function OSM variant affected hematopoietic stem cell function through changes to the bone marrow microenvironment (BMM).

## Beyond stem cell intrinsic defects in IBMFS

**Figure 1:**
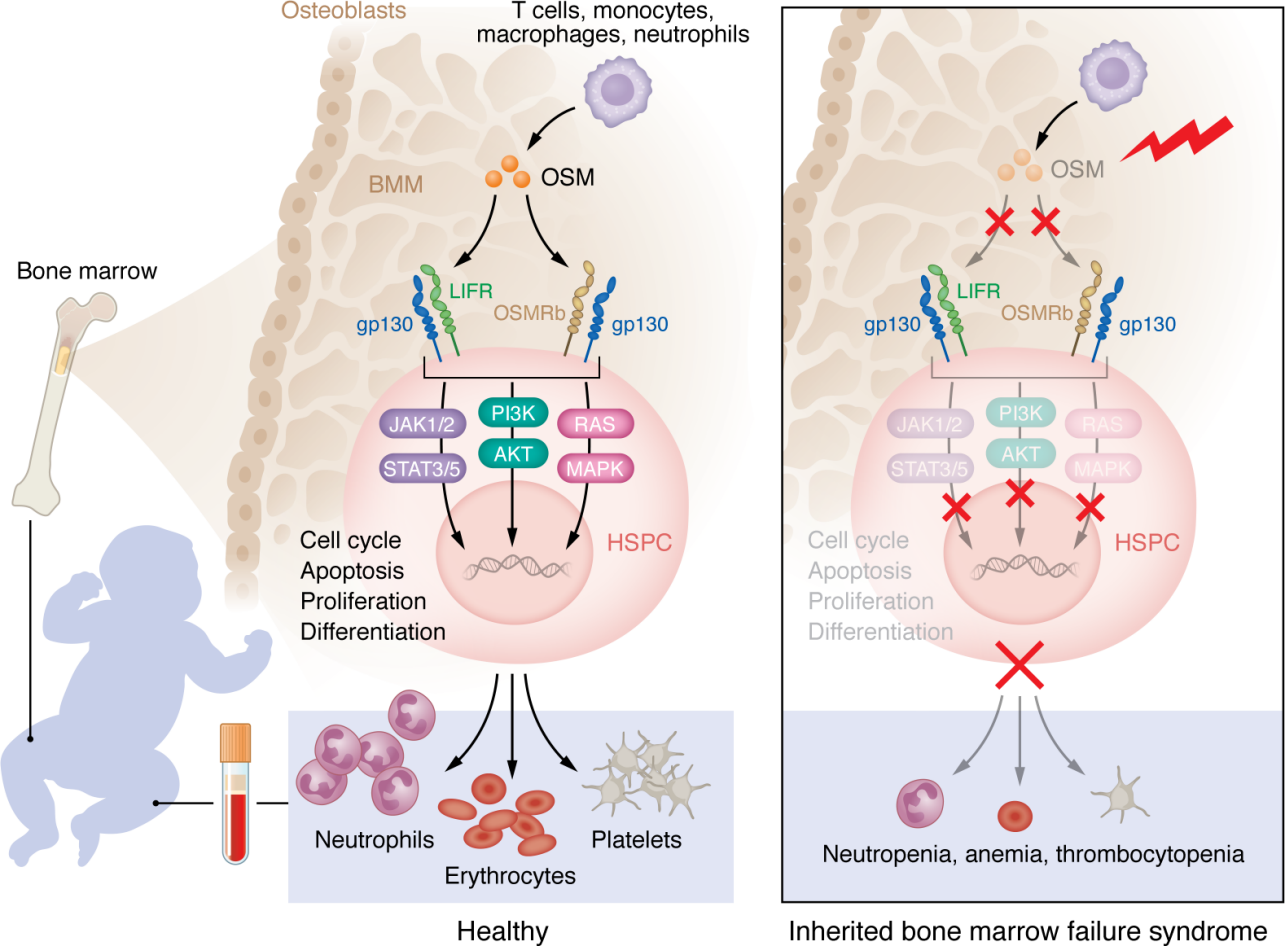
Autosomal recessive OSM deficiency causes an isolated phenotype of IBMFS through an alteration of the BMM. OSM is a pleiotropic cytokine, secreted by activated immune cells such as T cells, monocytes, macrophages, and neutrophils. Upon binding to its receptors, composed of gp130 and LIFR and/or gp130 and OSMRb, which are widely expressed in hematopoietic and nonhematopoietic tissue, OSM activates the JAK/STAT, the RAS/MAPK, and the PI3K/AKT signaling pathways. Garrigue, Kermasson, and colleagues (1) demonstrated how a homozygous LoF mutation, found in three children from a consanguineous family, leads to the production of a neopeptide, impeding the interaction with both OSM receptors on HSPCs, which ultimately underlies an IBMFS characterized by profound anemia, neutropenia, and thrombocytopenia. This discovery demonstrates that genetic causes of IBMFS extend beyond HSPC intrinsic defects, as alterations of the BMM and inflammatory cytokines can play a pathogenic role in the development of IBMFS.

In this issue of the *JCI*, Garrigue, Kermasson, and colleagues demonstrate that human oncostatin M (OSM) deficiency underlies profound anemia, thrombocytopenia, and neutropenia, unraveling another inherited bone marrow failure syndrome (IBMFS) (1). Using a combination of whole-genome homozygosity mapping (WGHM) and whole-exome sequencing (WES), the group identified a homozygous loss-of-function (LoF) variant in three children from a consanguineous family presenting with a particular phenotype of isolated pancytopenia (1) (Figure 1). IBMFSs represent a heterogenous group of rare monogenic disorders characterized by a progressive mono-, bi-, or trilineage cytopenia, often associated with nonhematological manifestations (2, 3). Known genetic causes of IBMFS comprise deleterious germline variants affecting DNA repair, telomere maintenance, and ribosome biogenesis and function, processes which are essential for the self-renewal of hematopoietic stem and progenitor cells (HSPCs) (4). Finally, defects in erythromyeloid differentiation, metabolism, and HSPC homeostasis can also underlie IBFMS. In IBMFSs such as Fanconi anemia, Diamond-Blackfan syndrome, dyskeratosis congenita, constitutional thrombocytopenia, and congenital neutropenia, different stresses elicit a p53-dependent growth arrest, resulting in an accelerated apoptosis of HSPCs (5).

Garrigue, Kermasson, and colleagues expand the etiology of IBMFS, demonstrating that next to intrinsic genetic defects affecting HSPCs, genetically defined alterations of the bone marrow microenvironment (BMM) may cause an IBMFS (1). Indeed, patients’ HSPCs differentiated normally into erythroblasts and thrombocytes in vitro, suggesting that OSM deficiency hampers maturation after erythroblast and megakaryocyte differentiation in an HSPC-intrinsic way, or that it alters the BMM. This hypothesis was supported by the normal colony-forming assay results and mega-cult assays using patient cells and previously by decreases in erythroid and megakaryocytic cells following transfer of bone marrow cells from WT mice to OSM receptor-deficient (OSMR-deficient) mice (1, 6). Furthermore, the Garrigue et al. findings support the emerging concept that sustained or aberrant exposure to inflammatory signals, force repeated HSC cycling, which can result in HSC exhaustion and hematopoietic failure (4).

## The value of exploring rare pathways in IBMFS

The three patients described by Garrigue, Kermasson, and colleagues were affected by an isolated IBMFS. The disease initially manifested between the ages of three months and six months. One sibling presented with anemia, thrombocytopenia, and neutropenia, whereas the two others presented with anemia and gradually developed thrombocytopenia and/or neutropenia over the following years. Dyserythropoiesis and dysgranulopoiesis became apparent upon cytological bone marrow analysis of two siblings by the age of seven to nine-and-a-half years, and reduced cellular density in one patient by the age of 18 years (1). Using WGHM and WES, the authors identified a homozygous one base-pair insertion in *OSM*, which segregated with the phenotype. OSM is a pleiotropic cytokine, belonging to the IL-6 subfamily, which has the particularity of signaling through two types of OSM receptors (OSMRs) that are widely expressed in hematopoietic and nonhematopoietic cells (7). OSM is secreted by activated immune cells, such as T cells, monocytes, macrophages, dendritic cells. and neutrophils. Upon binding to its receptors, OSM activates the JAK/STAT, the RAS/MAPK, and the PI3K/AKT signaling pathways. OSM was initially discovered due to its growth-inhibitory effect on tumor cell lines, which led to its naming (7). Meanwhile, it has become evident that OSM has pleiotropic effects, not only on cell proliferation but also on the regulation of inflammation, hematopoiesis, bone remodeling, and neuroprotection (8). Garrigue, Kermasson, and colleagues demonstrate that the homozygous *OSM* variant, detected in their patients, leads to a frameshift in the C-terminal part of OSM, resulting in a neopeptide. Notably, this neopeptide impeded interaction with the leukemia inhibitory factor receptor (LIFR) and the OSMR subunits, as confirmed in patient cells and CRISPR/Cas9 engineered UT7 cells. Modeling this loss of OSM signaling in splice-morpholino–treated zebrafish unveiled defective erythrocyte and neutrophil production (1). However, the morpholino zebrafish were not thrombocytopenic, pointing to the different thrombocyte development pathways in mammals versus zebrafish. This genetic discovery supports the use of next-generation sequencing to discover germline variants in patients with undefined and isolated IBMFS (5). Since IBMFS predispose to malignant transformation, they may also be discovered in patients affected by aplastic anemia, myelodysplastic syndrome (MDS), or malignancy over a range of ages (9).

## The mysteries of OSM unraveled

Abnormal levels of OSM play a crucial role in a multitude of inflammatory diseases. However, since OSM exerts various regulatory effects on inflammation, it may have opposing effects in different diseases. In humans, excessive OSM has been shown to drive inflammation in conditions such as inflammatory bowel disease, multiple sclerosis, rheumatoid arthritis, systemic sclerosis, and chronic inflammatory lung disease (7, 10–12). For a subset of these diseases, clinical trials with monoclonal antibodies directed against OSM are ongoing and small molecule drugs targeting OSM and OSMR are being developed (7, 8). On the other hand, OSM-deficient mice are affected by a reduced hematopoietic progenitor activity in the bone marrow and have a massive accumulation of apoptotic thymocytes with the production of autoantibodies (13).

Human disease linked to genetic OSM deficiency was not previously described. Biallelic LoF mutations in LIFR cause Stüve-Wiedemann syndrome (OMIM #601559), characterized by neonatal skeletal dysplasia, feeding difficulties, respiratory distress, and hyperthermia; extended Stüve-Wiedemann syndrome (OMIM #619751) is caused by biallelic deleterious variants in the gene encoding gp130, showing that gp130 deficiency is mostly due to LIFR signaling deficiency. Cytopenia has been very rarely reported in this condition. Also, IL31RA deficiency and OSMRb deficiency both result in primary localized cutaneous amyloidosis (OMIM #613955 and #105250) (1). Thus, the careful dissection of OSM deficiency as an inborn error unveils a rather limited effect, at least of this single variant causing a LoF in OSM. Nevertheless, the description of OSM deficiency suggests that therapeutic strategies addressing inflammatory disease with complete OSM blockage could result in adverse outcomes. Indeed, the human model of OSM deficiency, as described by Garrigue et al., is that of a progressive IBMFS. Although the patients had almost no extrahematopoietic manifestations, surprisingly, these symptoms may appear with age, and it is possible that the description of additional patients with other or identical genetic causes of OSM deficiency will broaden the phenotype over time.

## The quest for new genetic causes of IBMFS

The discovery of new causes of IBMFS, such as the OSM deficiency described in this report by Garrigue, Kermasson, and colleagues, has demonstrated that these conditions might not be as rare as previously thought (1). Next-generation sequencing enables broadening the spectrum of known germline variants causing IBMFS, and functional validation is needed to fully grasp the pathophysiology, to comprehend the natural evolution of these disorders, and ultimately to guide precision medicine. In fact, bone marrow failure has been classified as one of the presenting phenotypes of inborn errors of immunity (IEI) (14, 15). Recent discoveries of genetic causes of IBMFS with associated immune and/or syndromic features include deleterious variants in *SNM1B*, *DUT*, and *RAD50* (16–18). Recent additions also include (a) LoF mutations in *MECOM* underlying severe neonatal aplastic anemia without bone marrow dysplasia, radioulnar synostosis, and cardiac abnormalities, necessitating HSCT within the first year of life, (b) homozygous variants in *ERCC6L2* causing a mild pancytopenia with possible MDS, and (c) IBMFS due to variants in *SAMD9* and *SAMD9L,* in which hematopoietic somatic mosaicism can lead to attenuated bone marrow failure, denoting the need for HSCT (5, 19). On the other hand, for some less well-known causes of IBMFS, such as ADA2 deficiency, the precise pathological mechanism of bone marrow failure remains elusive and HSCT remains the only curative option until now (20, 21). Indeed, whereas TNF inhibition is mostly effective in treating the vasculitis in these patients, the bone marrow failure does not respond to it (22). The discovery of human autosomal recessive (AR) OSM deficiency underscores the importance of the BMM in the development of bone marrow failure and opens new avenues in the study of the pathophysiology of this and other ill-explained IBMFS.

## Discovering the unwritten chapters of IBMFS

The discovery of OSM deficiency as an underlying cause of IBMFS by Garrigue, Kermasson, and colleagues demonstrates that genetic causes of IBMFS extend beyond HSPC intrinsic defects, as alterations of the BMM can also result in IBMFS, and highlights inflammatory cytokines as playing a pathogenic role in the development of IBMFS. Unravelling the underlying pathophysiology of IBMFS is primordial to guide precision medicine and treatment. Although allogeneic hematopoietic stem cell transplantation (HSCT) remains a curative option for the hematological manifestations of IBMFS and provides malignancy prevention, its extrahematopoietic manifestations of IBMFS necessitate a careful risk-benefit evaluation. Novel therapeutic options might target the BMM or explore treatment alternatives beyond transplantation (23). Finally, the insights gained from OSM deficiency should prompt caution regarding the use of therapies inhibiting OSM or OSMR in common inflammatory diseases.

## Version 1 03/17/2025

Electronic publication
